# Existing maternal obesity guidelines may increase inequalities between ethnic groups: a national epidemiological study of 502,474 births in England

**DOI:** 10.1186/1471-2393-12-156

**Published:** 2012-12-18

**Authors:** Nicola Heslehurst, Naveed Sattar, Daghni Rajasingam, John Wilkinson, Carolyn D Summerbell, Judith Rankin

**Affiliations:** 1 Institute of Health & Society, Newcastle University, Newcastle upon Tyne, UK; 2 Faculty of Medicine, University of Glasgow, Glasgow, UK; 3 Obstetrics, Guys and St Thomas’ Hospital NHS Foundation Trust Hospital, London, UK; 4 School of Medicine, Pharmacy and Health, Durham University, England, UK

**Keywords:** Obesity, Pregnancy, Epidemiology, Inequalities, Ethnic group, Asian, Guidelines, Body Mass Index (BMI)

## Abstract

**Background:**

Asians are at increased risk of morbidity at a lower body mass index (BMI) than European Whites, particularly relating to metabolic risk. UK maternal obesity guidelines use general population BMI criteria to define obesity, which do not represent the risk of morbidity among Asian populations. This study compares incidence of first trimester obesity using Asian-specific and general population BMI criteria.

**Method:**

A retrospective epidemiological study of 502,474 births between 1995 and 2007, from 34 maternity units across England. Data analyses included a comparison of trends over time between ethnic groups using Asian-specific and general population BMI criteria. Logistic regression estimated odds ratios for first trimester obesity among ethnic groups following adjustment for population demographics.

**Results:**

Black and South Asian women have a higher incidence of first trimester obesity compared with White women. This is most pronounced for Pakistani women following adjustment for population structure (OR 2.19, 95% C.I. 2.08, 2.31). There is a twofold increase in the proportion of South Asian women classified as obese when using the Asian-specific BMI criteria rather than general population BMI criteria. The incidence of obesity among Black women is increasing at the most rapid rate over time (p=0.01).

**Conclusion:**

The twofold increase in maternal obesity among South Asians when using Asian-specific BMI criteria highlights inequalities among pregnant women. A large proportion of South Asian women are potentially being wrongly assigned to low risk care using current UK guidelines to classify obesity and determine care requirements. Further research is required to identify if there is any improvement in pregnancy outcomes if Asian-specific BMI criteria are utilised in the clinical management of maternal obesity to ensure the best quality of care is provided for women irrespective of ethnicity.

## Background

Maternal obesity poses significant and serious health risks to mothers and their babies [1-4], as well as having a major impact on maternity services for the prevention and management of these risks [5-7]. The serious implications of maternal obesity have led to an increasing inclusion in UK national guidelines and reports [1,2,8-12], as well as international guidelines [13-18].

There is an absence of pregnancy-specific body mass index (BMI) criteria to define maternal obesity. Research, guidelines, and clinical practice utilise the World Health Organisation (WHO) general population BMI criteria to define first trimester obesity [19]. These BMI criteria were set by the WHO in 1993 to define differential international categories of obesity to reflect the risk for cardiovascular disease and type 2 diabetes (BMI≥30kg/m^2^ for obesity, and ≥40kg/m^2^ for morbid obesity). These were updated in 1997 to include severe obesity (BMI 35.0-39.9kg/m^2^) [20]. Emerging evidence of an increased susceptibility to the metabolic effects of adiposity among Asian populations when compared with European Whites of a similar BMI led to further revisions by a WHO expert consultation [20]. There is clear evidence that South Asians in particular have a greater risk of diabetes in the BMI range considered ideal for European White populations. A comprehensive review identified the excess risk to be, in part, linked to greater total fat mass in South Asians, with consequently more rapid and earlier accumulation of fat in the key organs linked to diabetes (such as muscle and the liver) [21]. There is also evidence that South Asian populations have a lesser ability to metabolise fat versus carbohydrates which may increase their susceptibility to associated morbidities [22]. Pre-pregnancy BMI also has a significantly greater effect on insulin resistance among Asian women compared with White women; therefore ethnicity modulates the effects of obesity on insulin resistance in pregnancy [23]. The WHO consultation identified that due to the increased risk of morbidity among Asian populations at a lower BMI than the general population, the criteria for Asian populations should be reduced to reflect this risk (Table 1) [20].

**Table 1:** A comparison of the WHO BMI classifications for the general population and for Asian populations

	**WHO general population BMI classifications**	**WHO asian BMI classifications**
Underweight	<18.5kg/m^2^	<18.5kg/m^2^
Ideal	18.5-24.9kg/m^2^	18.5-23kg/m^2^
Overweight	25.0-29.9kg/m^2^	23-27.5kg/m^2^
Obese	≥30kg/m^2^	>27.5kg/m^2^

The existing literature on the combined impact of maternal BMI status, ethnic group, and pregnancy outcome is minimal. Research in the USA has identified disparities in obstetric outcomes between ethnic groups, particularly for African American and Hispanic women, including pre-term labour, pre-eclampsia, gestational diabetes, pregnancy induced hypertension and increased caesarean section rate among others [24-28]. Research has also identified disparities in obstetric risk among Asian women, including third and fourth degree lacerations, puerperal infection, gestational diabetes, antenatal and postpartum haemorrhage, pre-eclampsia and hypertensive disorders [24,25,28-31]. The inter-relationship between ethnic group and obesity was also found to increase the risk of adverse pregnancy outcomes such as macrosomia, caesarean delivery, and operative vaginal delivery, with variable effect between ethnic groups depending on the outcome being assessed [25,26]. However, these studies did not use the Asian-specific criteria to define maternal weight status.

There is also a paucity of comparative research in UK populations. The Centre for Maternal and Child Enquires (CMACE) reported that the relative risk (RR) of maternal mortality among Black women was significantly increased compared with White women (RR 3.29, 95% confidence interval (CI) 2.28–4.75) [1]. This was most pronounced in Black African women (RR 3.85, 95% CI 2.53–5.88), and Black Caribbean women (RR 3.75, 95% CI 1.84–7.62). Risk of maternal mortality among South Asian women was also increased, although not significant (RR 1.44, 95% CI 0.93–2.23). Variations in mortality risk were also present between South Asian subgroups, with Pakistani women having the highest risk of pregnancy-related mortality. CMACE also reported an increased rate of stillbirth and neonatal mortality among Black and Asian women compared with White women [1]. However, these analyses of ethnic group and maternal and perinatal mortality risk did not account for the combined effect of BMI.

UK national datasets have identified a twofold increase in first trimester obesity over two decades, increased risk of obesity-related adverse pregnancy outcomes, and associations with socio-demographic inequalities [12,32,33]. Obesity associated obstetric risk identified in the datasets included induction of labour, caesarean delivery, and intensive care use among others [12,33]. However, these datasets did not differentiate between general population and Asian specific BMI criteria, or account for any combined effect of BMI and ethnic group. In addition, current UK guidelines for the clinical management of maternal obesity [9,10] and weight management [11] do not differentiate between the BMI criteria for the general population and Asian populations.

Attempts to rectify ethnic-related disparities in obstetric outcomes should begin with an accurate account of the epidemiology [27]. Given the strong evidence of greater rates of gestational diabetes in Asian populations with lower or similar BMIs to White populations [23,26,34], and the international evidence of the combined effects of ethnic group and obesity on obstetric risk, it is important to report maternal obesity incidence using the Asian-specific BMI criteria [20]. This study compares ethnic group trends in maternal BMI using the WHO Asian-specific and general population BMI criteria in a national dataset for England.

## Methods

### Identification of eligible maternity units

A postal survey was carried out among all NHS maternity services in England (n=243) between 2006 and 2007 to establish the routine data collection practice among NHS Trusts. The survey was designed in accordance with the principles of the Tailored Design Method (TDM) which is based on the social exchange theory of human behaviour where the response to surveys is reflective of the perceived benefits and costs to the individual [35]. Dillman recommends that the development of survey procedures should create respondent trust, perceptions of increased reward and reduced costs for being a respondent, taking into account the features of the survey situation, with the goal to reduce survey error by increasing response rate. Applying the principles of the TDM included personalising the survey with the respondents details (head of midwifery and lead obstetrician for each maternity unit), providing multiple methods of responding (post, email, or web-based questionnaire), providing a pre-paid return envelope, keeping the questionnaire short (one A4 page), using colour and institutional logos, and following up non-response (up to 2 reminders for non-responders). The survey questions asked whether maternity units routinely collected data electronically, the year that electronic data collection commenced, and routine electronic data collection of the following data items: date of booking (1^st^ antenatal contact with maternity services), stage of pregnancy at booking, maternal age, parity, weight, height, BMI, ethnic group, date of delivery, gestational age at delivery (to calculate gestation at booking), postcode (to define deprivation), and maternal employment. The survey data were used to identify potentially eligible maternity units which routinely collected the required data for the study in an electronic format. All eligible units were invited to participate in the study.

### Data coding

Ethnic groups were defined using standard national census categories in order to be comparable with existing general population datasets in the UK [36]:

● White (White British, White Irish, and other White)

● South Asian (Bangladeshi, Indian, Pakistani, and other South Asian)

● Black (Black Caribbean, Black African, and other Black)

● Mixed (White and Black African, White and Black Caribbean, White and Asian, and other Mixed)

● Chinese or Other Ethnic Group (Chinese and all Other Ethnic Groups)

Throughout this paper, the term Asian refers to women from the South Asian, Chinese, and Asian women within the Other Ethnic Group UK census categories. The term South Asian is used specifically to refer to women from Bangladeshi, Indian, Pakistani, and other South Asian populations.

Maternal BMI status in the first trimester was defined using the general population BMI criteria for all non-Asian ethnic groups [19]. The BMI data for all Asian women were double coded to the general population criteria, as well as the Asian-specific BMI criteria [20] to allow comparison of trends using both classification reference criteria.

Maternal deprivation was defined based on the IMD 2007 reference criteria. The IMD ranks postcodes based on the area’s deprivation levels of income, employment, health, disability, education, skills and training, barriers to housing and services, living environment, and crime [37]. The rank of deprivation ranges from 1 (most deprived) to 32,482 (least deprived). The quintiles used in this study were calculated as five equal proportions of the rank of deprivation.

### Data analysis

Data did not require adjustment for age over time as there was no significant change in population age over time (range in mean age: 28 years, SD 5, and 29 years, SD 6). Maternal BMI data were adjusted for naturally incurred gestational weight gain among women who were late bookers (after their first trimester). Published data on BMI change per gestational week were used to estimate the first trimester BMI for late bookers [38]. White ethnic group was the majority population and therefore used as the reference group. Trends in first trimester obesity for individual ethnic groups were explored by comparing the linear component of the incidence slopes over time using 95% CIs. Logistic regression was carried out to analyse the relationship between obese BMI and ethnic group, adjusting for age, parity, employment, and deprivation quintile. Multicolinearity tests were carried out using linear regression diagnostics and Pearson’s r correlation tests. No multicolinearity was present between the predictor variable and any other variable, and therefore all were included in the final regression model.

Multicentre research ethics committee approval was obtained (Sunderland NHS MREC), along with research and development (R&D) approval for each NHS Trust included in the study (n=24).

## Results

The survey response was 89% (n=217). Of the respondents, 51 maternity units did not collect data electronically and 31 units with electronic data collection did not electronically record anthropometric data. Invitations to participate were sent to the remaining 135 maternity units. Seven did not want to participate, 70 did not respond, and 58 wanted to participate. Twelve maternity units were excluded as they incorrectly reported electronic data collection in the survey (primarily employment data were missing), five withdrew due to inadequate recording of BMI, the R&D approval process took over one year and was not approved at the time of data analysis in two units (one NHS trust), and two units withdrew due to staffing issues. Data were provided for 37 maternity units, a further three units were excluded from the analysis as their ethnic groups data were poor quality (high proportion of missing data). Anonymised electronic datasets for 34 maternity units in 24 NHS trusts were included in the analysis.

The dataset included 552,303 births between 1995 and 2007 across England. The dataset was comparable with the socio-demographics of women of childbearing age using England census [39], and IMD comparison data [37] (Table 2). Following exclusion of missing ethnic group data (9%), data for 502,474 deliveries remained. The excluded group was similar to the included group in mean age (29.5 years, SD 6.0 and 28.8 years, SD 6.0) and mean BMI (24.3kg/m^2^, SD 4.9 and 24.7 kg/m^2^, SD 5.2). Women who were excluded due to missing ethnic group were more likely to be residing in areas of least deprivation compared with women who were included (30.3% and 20.7%), and less likely to be residing in areas of highest deprivation compared with women who were included (15.9% and 23.3%).

**Table 2:** A socio-demographic comparison of the study population and women of childbearing age in the general population

	**Proportion of pregnant women in the study population (%)**	**Proportion of women of childbearing age in england (%)**
Ethnicity (women aged 15–44)*		
White	82.7	88.4
Mixed	1.2	1.4
Asian or Asian British	9.5	5.6
Black or Black British	4.4	3.2
Chinese/Other Ethnic Group	2.2	1.4
Employment Status (women aged 16–44)*		
Paid Employment	64.5	62.8
No Paid Employment	32.2	24.1
Education	3.2	13.1
**Deprivation Quintile (women aged 15–44)^**		
Most Deprived 1	23.3	21.5
2	20.4	21.0
3	18.5	19.9
4	17.2	19.0
Least Deprived 5	20.7	18.5

* 2001 Census Data.

^ 2007 IMD Data.

The included study population was predominantly aged 21–30, White, employed, had a parity of 0 or 1, mean BMI of 24.7kg/m^2^, mean booking gestation of 14 weeks, and an even distribution across deprivation quintiles (Table 3).

**Table 3:** Population characteristics of the pregnant women included, between 1995 and 2007 in England

	**Total**	**Underweight**^**a**^	**Ideal**^**b**^	**Overweight**^**c**^	**Obese**^**d**^
**BMI, mean (SD)**	24.7 (5.2)	17.3 (1.0)	21.9 (1.7)	26.8 (1.6)	34.1 (4.5)
**BMI, median (interquartile range)**^**e**^	23.7 (5.9)	17.6 (1.3)	22.0 (2.8)	26.9 (2.4)	33.3 (5.1)
**Gestational Age at Booking in Weeks, mean (SD)**	13.9 (6.5)	19.4 (10.5)	13.8 (6.2)	13.5 (5.9)	13.1 (5.6)
**Booked after 1**^**st**^**Trimester, n (%)**	204,355 (40.7)	5,869 (37.1)	108,930 (39.5)	56,965 (42.7)	32,591 (42.3)
**Parity Group, n (%)**					
0	175,826 (37.8)	10,241 (43.2)	102,727 (40.5)	42,265 (35.3)	20,593 (30.7)
1	174,980 (37.7)	8,396 (35.4)	95,497 (37.6)	45,710 (38.1)	25,377 (37.8)
2	81,622 (17.6)	3,700 (15.6)	40,918 (16.1)	22,652 (18.9)	14,352 (21.4)
3 or more	32,172 (6.9)	1,385 (5.8)	14,770 (5.8)	9,256 (7.7)	6,761 (10.1)
**Age Group, n (%)**					
<20	51,227 (10.2)	5,324 (20.8)	31,066 (11.4)	9,986 (7.7)	4,851 (6.5)
21-30	243,128 (48.4)	13,496 (52.6)	129,160 (47.5)	62,807 (48.3)	37,665 (50.6)
31-40	198,598 (39.5)	6,566 (25.6)	107,276 (39.4)	54,462 (41.9)	30,294 (40.7)
>41	9,350 (1.9)	257 (1.0)	4,626 (1.7)	2,802 (2.2)	1,665 (2.2)
**Ethnic Group, n (%)**					
South Asian	47,867 (9.5)	3,903 (15.2)	18,396 (6.8)	15,752 (12.1)	9,816 (13.2)
Black	22,250 (4.4)	960 (3.7)	9,498 (3.5)	7,185 (5.5)	4,607 (6.2)
Mixed	5,797 (1.2)	418 (1.6)	3,274 (1.2)	1,362 (1.0)	743 (1.0)
Chinese or Other Ethnic Group	11,067 (2.2)	1,016 (4.0)	6,371 (2.3)	2,676 (2.1)	1,004 (1.3)
White	415,493 (82.7)	19,353 (75.5)	234,676 (86.2)	103,130 (79.3)	58,334 (78.3)
**Employment, n (%)**					
Not Employed	39,903 (11.0)	3,178 (17.6)	21,676 (11.0)	9,130 (9.8)	5,919 (11.0)
Housewife/Carer	76,584 (21.2)	4,864 (26.9)	37,596 (19.1)	20,278 (21.8)	13,846 (25.7)
Higher Education	7,397 (2.0)	501 (2.8)	4,134 (2.1)	1,800 (1.9)	962 (1.8)
School Age/Education under 18 years	4,318 (1.2)	543 (3.0)	2,981 (1.5)	589 (0.6)	205 (0.4)
Employed	233,264 (64.5)	9,009 (49.8)	130,191 (66.2)	61,154 (65.8)	32,910 (61.1)
**Deprivation Quintile, n (%)**					
1 Most Deprived	115,162 (23.3)	6,978 (27.8)	55,649 (20.8)	30,702 (23.9)	21,833 (29.7)
2	101,127 (20.4)	5,437 (21.6)	51,361 (19.2)	27,302 (21.3)	17,027 (23.1)
3	91,508 (18.5)	4,437 (17.6)	49,724 (18.5)	23,985 (18.7)	13,362 (18.2)
4	85,000 (17.2)	3,801 (15.1)	49,105 (18.3)	21,539 (16.8)	10,555 (14.3)
5 Least Deprived	102,454 (20.7)	4,492 (17.9)	62,311 (23.2)	24,826 (19.3)	10,825 (14.7)

^a^ Underweight BMI defined as <18.5 kg/m^2^ for all ethnic groups.

^b^ Ideal BMI defined as 18.5-22.9 kg/m^2^ for Asian populations, and 18.5-24.9 kg/m^2^ for non-Asian populations.

^c^ Overweight BMI defined as 23–27.4 kg/m^2^ for Asian populations, and 25.0-29.9 kg/m^2^ for non-Asian populations.

^d^ Obese BMI defined as >27.5 kg/m^2^ for Asian populations, and ≥30 kg/m^2^ for non-Asian populations.

^e^ BMI data were positively skewed therefore median and interquartile range also presented for comparison with the mean.

When using the general population criteria for obesity, 10.6% of South Asian women were classified as obese. However, this increased to 20.5% when using the WHO Asian-specific obesity criteria. Similarly, overweight increased from 25.7% to 32.9%. The ideal group decreased from 55.5% to 38.4%. Underweight remained constant at 8.2% as the criteria does not alter between general and Asian-specific populations. A similar pattern was observed among women of Asian origin in the Chinese or Other Ethnic Group category, albeit less substantial. Obesity increased from 7.7% to 9.1%; overweight increased from 20.6% to 24.2%; ideal weight decreased from 62.5% to 57.6%; and underweight remained constant at 9.2%.

**Figure 1:**
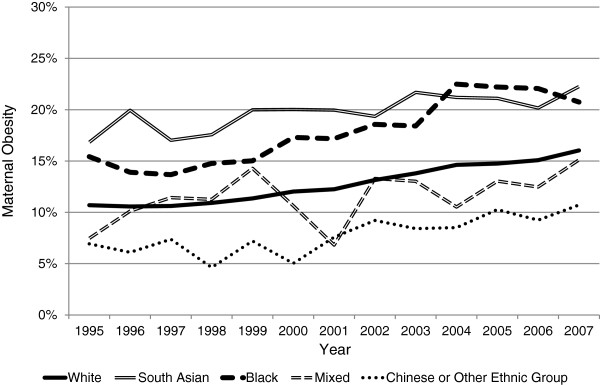
**A Comparison of First Trimester Obesity Incidence over Thirteen Years in England stratified by Maternal Ethnic Group.** Legend: Maternal obesity is classified as a BMI >27.5kg/m^2^ for Asian populations, and ≥30kg/m^2^ for non-Asian populations.

The remaining analyses used the Asian WHO BMI criteria for all Asian women, and the general population criteria for all non-Asian women. Maternal obesity increased over time among all ethnic groups; however there was significant variability (Figure 1). The incidence of obesity was significantly higher among South Asian and Black women compared with White women, and lower among women from the Chinese or Other Ethnic Group category. The comparison of the linear component of the incidence slopes of all ethnic groups with White women identified that the slope for Black women was significantly steeper than that for White women (difference between slopes=0.28, 95% CI 0.06 to 0.49, p=0.013). Therefore first trimester obesity among Black women is increasing at a more rapid rate over time than among White women. There was no significant difference in the linear component of the incidence slopes between White and South Asian women (difference between slopes=0.14, 95% CI −0.04 to 0.32, p=0.13), or women in the Chinese or Other Ethnic Group category (difference between slopes=0.11, 95% CI-0.09 to 0.31, p=0.25). The analyses could not be carried out for Mixed Ethnic Group due to the erratic nature of the slope.

**Figure 2:**
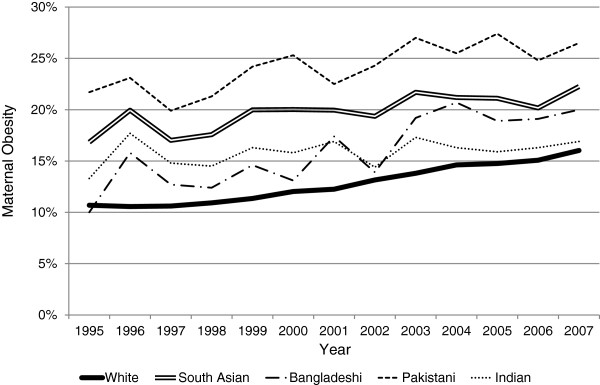
**A Comparison of First Trimester Maternal Obesity Incidence over Thirteen Years in England stratified for South Asian and White Ethnic Groups.** Legend: Maternal obesity is classified as a BMI >27.5kg/m^2^ for South Asian women, and ≥30kg/m^2^ for White women.

The incidence of obesity among the South Asian ethnic subgroups (Bangladeshi, Indian and Pakistani) was consistently higher than White women over the time period analysed (Figure 2). There are similar obesity trends between Bangladeshi and Indian women which are both increased compared with White women. However, the incidence of first trimester obesity among Pakistani women was consistently higher than Bangladeshi, Indian, and White women. When comparing the linear component of the incidence slopes of the White ethnic group with the South Asian subgroups, a significant difference was found between Indian and White women (difference between slopes=0.35, 95% CI 0.16 to 0.54, p=0.001). Therefore the incidence of obesity in Indian women is increasing significantly less rapidly than White women. There was no significant difference in rate between White and Pakistani women (difference between slopes=0.02, 95% CI −0.22 to 0.26, p=0.86), or Bangladeshi women (difference between slopes=−0.26, 95% CI −0.56 to 0.04, p=0.09). Subgroup analyses of Black women could not be carried out due to the low quality of data coding for this ethnic groups’ subgroups (frequent use of the code Other Black Ethnic Group rather than Black African or Black Caribbean).

The regression analyses identified that both Black and South Asian women were significantly more likely to be obese when compared with White women following adjustment for demographic variables with potential confounding (Table 4). Among the South Asian subgroups Bangladeshi, Indian, and Pakistani women all had a significantly increased odds of being obese compared with White women. This was most pronounced among Pakistani women, compared with Bangladeshi and Indian women. The odds of being obese were lowest among Chinese or Other Ethnic Group women.

**Table 4:** Adjusted odds ratios^a^ for maternal obesity^b^ stratified by Ethnic group in England, UK, 1995 to 2007

**Ethnic group**	**Odds ratio (95% CI)**
White	Reference
South Asian	1.72 (1.66-1.79)
Pakistani	2.19 (2.08-2.31)
Bangladeshi	1.15 (1.06-1.24)
Indian	1.49 (1.39-1.60)
Black	1.70 (1.62-1.78)
Mixed	0.79 (0.72-0.87)
Chinese or Other Ethnic Group	0.63 (0.58-0.69)

^a^ Adjustments included parity, age, IMD score, and employment.

^b^ Obese BMI defined as >27.5kg/m^2^ for Asian populations, and ≥30kg/m^2^ for non-Asian populations.

## Discussion

This is the first national maternal obesity dataset to be analysed within the UK or internationally using the WHO Asian-specific BMI criteria. Applying these criteria doubled the incidence of first trimester obesity among South Asian women compared with using the general population criteria for obesity. The data also identified that, following adjustments for potential confounding, the incidence of first trimester obesity is higher among South Asian and Black women compared with White women. Moreover, Pakistani women have the highest odds of being obese compared with any other ethnic group when using the Asian-specific BMI criteria. These findings are representative of obesity among women of ethnic groups in the general population, where Black and South Asian women have a significantly higher prevalence of obesity compared with White women [40].

Using inappropriate BMI criteria to determine population trends in obesity underestimates the incidence of maternal obesity among Asian populations, which has implications for health service planning, commissioning, and delivery. Using the internationally recognised ethnic group-specific BMI criteria provides a better estimate of trends and is more reflective of the risks associated with BMI among Asian populations [23,34,41], and the prevalence of obesity among ethnic groups in the general population in England [42].

### Healthcare policy and practice implications

Current UK guidelines which include maternal obesity recommendations do not differentiate between the use of BMI criteria for the general population and Asian-specific criteria [9-11]. Clinical and public health guidelines should be tailored to meet the specific mapped needs of the population. While the majority ethnic group within the UK is White, South Asians are the second largest ethnic group. In addition, South Asian women make up a significant proportion of the antenatal population in some UK regions, and are sometimes the majority population of women accessing antenatal services. Thus, national guidelines which do not differentiate between ethnic groups or use appropriate reference criteria for determining risk are not serving maternity service providers and patients in these geographical regions.

In addition to the physiological mechanisms which contribute to increased susceptibility of morbidity in Asian populations at lower BMIs (as previously discussed) [21,22], there are also ethnic group related inequalities in antenatal care which impact on the health of pregnant women and their babies. There are known inequalities in access to antenatal care among ethnic minority groups in the UK [1,43], as well as UK and international evidence of sub-standard care provision among ethnic groups resulting in increased morbidity and mortality [24,44,45]. Additional inequalities in maternal mortality in England may be culturally derived, and reflect sub-optimal social circumstances especially for women who have recently migrated to the UK [1]. However, an additional concern for policy and practice is the adequacy of using general population BMI criteria to determine obesity-related risk, and subsequent implementation of measures to prevent and manage risk such as additional monitoring, screening and lifestyle intervention. If the general population BMI criteria are systematically inadequate to determine risk among Asian women, then current guidelines would be recommending an incomplete risk assessment at booking for women from these ethnic groups. Incorrect assignation to low risk care would contribute to a poorer prognosis for the mother and her baby, and further accentuate health inequalities associated with ethnicity and pregnancy.

However, promoting evidence-based practice through the development of evidence-based guidelines is dependent on the existence of research to inform health care policy and decision making. Although there is international evidence of differential obstetric risk between ethnic groups (including Asian populations) [24-31] and the inter-relationship between obesity and ethnic groups [23,25,26], the evidence-base for South Asian populations is limited. The majority of international evidence is from USA populations where the combined Asian and Pacific Islanders (API) ethnic group category is not comparable with the discrete UK categories of South Asian and Chinese or Other Ethnic Group. As identified in our data, there is wide variation in maternal obesity incidence between South Asians and Asian women in the Chinese or Other Ethnic Group categories, even when using the appropriate BMI reference criteria. There is also evidence of disparities in risk within Asian sub-groups. Chu et al. [34] identified a significantly increased prevalence ratio (PR) of gestational diabetes among women in the API ethnic group compared with White women (PR 1.64, 95% CI 1.61-1.68). However, there were further significant differences within the API sub-groups, with Asian Indian women having the highest prevalence of gestational diabetes (PR 2.10, 95% CI 2.03-2.18), and Japanese women the lowest prevalence (PR 0.79, 95% CI 0.79-1.04). The authors posed several arguments for this variation between Asian Indian populations and other API sub-groups including differences in trends of overweight and obesity, central adiposity, and body fat percentage [34].

Therefore, despite there being an existing international evidence-base for ethnic groups, obesity and obstetric risk, it is not necessarily reflective of South Asians and has limited transferability to UK populations. There is also a lack of comparative UK research evidence. In addition, there is a lack of international or UK evidence of obstetric risk and the effectiveness of intervention using the WHO BMI criteria for Asian populations. Therefore the absence of ethnic group specific BMI criteria in current guidelines for maternal obesity is not surprising.

### Research implications

Due to increasing incidence of obesity in maternity populations, a better understanding of the combined effects of obesity and ethnic groups are required to inform guideline development and provide appropriate care for all populations [25]. Given that this study has identified that maternal obesity incidence doubles among South Asian populations when Asian-specific criteria are applied, there is an urgent need for further research to identify whether differential obesity criteria for Asian pregnant women better identifies those at risk. This is an important question given data from the Hyperglycaemia and Adverse Pregnancy Outcomes (HAPO) study [46] which may lead to lower defined glucose cut-points to define gestational diabetes, and linked evidence showing the benefits of lifestyle intervention in women with mild gestational diabetes [47]. In addition, the absence of research exploring UK-specific populations and the relationship between ethnic group and obesity associated obstetric outcomes needs to be addressed. In particular to identify if Asian women are at risk of obesity-associated obstetric complications at a lower BMI than the general population, such as the interacting risk of obesity and ethnicity on adverse pregnancy outcomes identified in international research [24-26].

### Study limitations and strengths

This study has several strengths including the large sample size, and the socio-demographic comparability with women of childbearing age in England. However, there are also several study limitations which predominantly relate to the use of routinely collected maternity data. There was inadequate coding of data relating to Black ethnic groups which limited the comparison of Black African and Caribbean ethnic sub-groups. Additionally, census coded ethnic group data does not represent the internal diversity of ethnic groups such as migrant status, religion, and cultural beliefs. However, the use of census coding does allow comparison with existing UK population data, and any international data which uses this method of ethnic group categorisation. This dataset also had a substantial proportion of women who booked late, after their first trimester. This may have introduced a systematic bias towards overestimation of overweight and obesity due to the BMI calculation incorporating naturally incurred pregnancy weight gain. Therefore the BMI for late bookers was adjusted using population estimates of change in BMI at each week of gestation to account for change in BMI beyond the first trimester. However, if this adjustment model was inaccurate it may have produced under- or over-estimates of booking BMI among populations who are more likely to book late, including ethnic minority groups.

## Conclusion

The current status of the evidence-base in relation to maternal BMI, ethnic group, and pregnancy-related risk is inadequate to fully inform the development of guidelines which are applicable to the characteristics of the whole UK population. Investment in developing this evidence-base is required to ensure that guidelines are more representative, and that the best quality of care is provided to all women irrespective of ethnicity.

## Competing interests

The authors declare that there are no conflicts of interest.

## Authors’ contributions

All authors made substantive intellectual contributions to the design of the research, interpretation of the data, drafting and revising the manuscripts and approved the final version to be published. NH carried out the data acquisition and analyses.

## Pre-publication history

The pre-publication history for this paper can be accessed here:

http://www.biomedcentral.com/1471-2393/12/156/prepub
